# Electroacupuncture intervention of visceral hypersensitivity is involved in PAR-2-activation and CGRP-release in the spinal cord

**DOI:** 10.1038/s41598-020-67702-2

**Published:** 2020-07-07

**Authors:** Manoj K. Shah, Yi Ding, Juan Wan, Habibullah Janyaro, Adnan Hassan Tahir, Vitaly Vodyanoy, Ming-Xing Ding

**Affiliations:** 1 College of Veterinary Medicine, Huazhong Agricultural University, 1 Shizishan Street, Hongshan District, Wuhan, 430070 Hubei Province People’s Republic of China; 2 Department of Surgery and Pharmacology, Agriculture and Forestry University, Bharatpur, Nepal; 3 Department of Anatomy, Physiology and Pharmacology, Auburn University, Auburn, AL USA

**Keywords:** Neuroscience, Gastroenterology, Medical research

## Abstract

Electroacupuncture (EA) relieves visceral hypersensitivity (VH) with underlying inflammatory bowel diseases. However, the mechanism by which EA treats ileitis-induced VH is not clearly known. To assess the effects of EA on ileitis-induced VH and confirm whether EA attenuates VH through spinal PAR-2 activation and CGRP release, goats received an injection of 2,4,6-trinitro-benzenesulfonic-acid (TNBS) solution into the ileal wall. TNBS-injected goats were allocated into VH, Sham acupuncture (Sham-A) and EA groups, while goats treated with saline instead of TNBS solution were used as the control. Goats in EA group received EA at bilateral Hou-San-Li acupoints for 0.5 h at 7 days and thereafter repeated every 3 days for 6 times. Goats in the Sham-A group were inserted with needles for 0.5 h at the aforementioned acupoints without any hand manipulation and electric stimulation. Visceromotor responses to colorectal distension, an indicator of VH, were recorded by electromyography. The terminal ileum and thoracic spinal cord (T_11_) were sampled for evaluating ileitis at days 7 and 22, and distribution and expression-levels of PAR-2, CGRP and c-Fos on day 22. TNBS-treated-goats exhibited apparent transmural-ileitis on day 7, microscopically low-grade ileitis on day 22 and VH at days 7–22. Goats of Sham-A, VH or EA group showed higher (*P* < 0.01) VH at days 7–22 than the Control-goats. EA-treated goats exhibited lower (*P* < 0.01) VH as compared with Sham-A or VH group. Immunoreactive-cells and expression-levels of spinal PAR-2, CGRP and c-Fos in the EA group were greater (*P* < 0.01) than those in the Control group, but less (*P* < 0.01) than those in Sham-A and VH groups on day 22. Downregulation of spinal PAR-2 and CGRP levels by EA attenuates the ileitis and resultant VH.

## Introduction

Inflammatory bowel diseases (IBDs) are chronic, immune-mediated and remittent-relapsing gastrointestinal inflammation. Crohn’s disease (CD) and ulcerative colitis (UC) are the predominant clinical types of IBDs and their resultant symptoms include diarrhea, nausea, weight loss, abdominal pain and visceral hypersensitivity (VH)^1,2^. There are two-thirds of IBD patients suffering from VH during their acute flares and remission^3,4^. The persistent nature of VH impairs the patient’s quality of life and consumes huge medical resources throughout the world. The environmental stress, inflammation and traumatic injuries trigger the development of VH. However, its underlying mechanism is not completely understood. A G-protein coupled receptor, PAR-2, is activated due to proteolytic cleavage of trypsin, tryptase and cathepsin-S^5–7^. Over the years, studies have reported that the PAR-2 activation on resident mast cells, neutrophils or macrophages initiates for the release of inflammatory mediators, i.e., cytokines, chemokines and prostaglandins, which triggers the production of neurogenic mediators, i.e., substance-P (SP) and calcitonin gene-related peptide (CGRP) from the enteric neurons as well as afferent nerves to elicit eventually the VH^8–10^. Cenac et al.^11^ infused the suboptimal inflammatory dose of PAR-2-agonists into the colon of rats and reported persistently prolonged VH in  colorectus-distended rats. The direct involvement of PAR-2 activation in  enteric neurons for developing the VH is still debatable. However, the infusion of the fecal supernatant of the patients with irritable bowel syndrome into the colon of healthy mice resulted in higher intestinal permeability, mucosal inflammation and subsequent VH through a PAR-2 activation mechanism^12–14^. These studies clearly indicate that activated PAR-2 in the intestines facilitates VH.

Central sensitization seems to have a crucial role both in the induction and maintenance of VH. It is essential to note the importance of cathepsin-S in the activation of nociceptive neurons of the spinal cord through the PAR-2-signalling mechanism, which intensifies and maintains chronic visceral pain^15,16^. Therefore, the role of spinal PAR-2 in VH could be an interesting area for the researchers. It is observed that the huge number of PAR-2-expressed primary spinal afferent neurons coexpress proinflammatory CGRP^17^. Activation of PAR-2 by its agonists enhances potassium chloride ion and potentiates the capsaicin-induced release of CGRP in sensory neurons^17,18^. The roles of CGRP in VH have been confirmed with CGRP-knockout and CGRP-receptor antagonists in rodents^19–22^. These findings demonstrate that spinal PAR-2 activation and the release of PAR-2-mediated CGRP contribute to the development of VH. Therefore, the blockade or inhibition of either of PAR-2 and CGRP or both could be a novel approach for managing VH in patients.

Nowadays, VH is mainly treated with steroidal/nonsteroidal anti-inflammatory drugs, opioids, or immunosuppressants, which result in sub-optimal analgesic effects, side effects or drug addiction. Therefore the availability of a novel therapeutic method is still imperative. Acupuncture, a traditional therapy, has been used substantially in China since the ancient period. Recently, EA, a modern vision of traditional acupuncture, has been proven efficient in treating the various pains, including chronic visceral pain in patients with gastrointestinal disorders^23–26^. Several studies have confirmed the superior effects of EA on visceral pain over the traditional acupuncture and EA at non-acupoint in rats^27^ and in human patients^28–31^. Wan et al.^32^ demonstrated EA abolished pain behavior manifestation and abdominal electromyographic values in response to colorectal distension (CRD) in VH-induced goats. Earlier studies^23,28^ also reported that the EA reduced abdominal EMG values in colorectal distended rats and mice. These findings indicate that EA might be the promising complementary therapy for the treatment of VH. Sun et al.^33^ described that EA attenuates VH in diarrheic-irritable bowel syndrome (IBS) model of rats through suppressing CGRP expression in the spinal cord. However, whether EA relieves ileitis-induced VH through suppressing the spinal PAR-2 activation or PAR-2-mediated CGRP release is not reported.

The c-Fos, an important early gene, is produced very quickly but transiently expressed in cells in response to specific stimulation. It is considered as a valuable marker for the identification of stimulated nuclei or region in the central nervous system (CNS)^34,35^. In addition, the continuous or subsequent nociceptive stimuli increase spinal c-Fos expression, which contributes to the generation of a pain state partly because of adaptive spinal response^34,36^. Therefore, c-Fos is useful for evaluating the effects and acting sites of active substances or treatments in the CNS.

EA in combination with an analgesic reduces the dosage of the analgesic drug by 40%-46%, 50%, and over 75% in humans, rats, and goats, respectively, with similar analgesic effects attained by the administration of the analgesic alone^37–39^. These findings clearly indicate that EA induces a superior analgesic effect in goats than in humans or rats, and thus the goats could be a preferred model animal to explore the precise mechanism of EA-induced analgesia. In this study, ileitis-induced VH has provoked through injection of 2,4,6-trinitro-benzenesulfonic-acid (TNBS)/ethanol solution in the ileal wall of goats. EA was used to stimulate the bilateral Hou-San-Li (ST36) acupoints of goats with the ileitis-induced VH goats firstly on day 7 and thereafter at an interval of 3 days up to 6 times. VH was evaluated with the visceromotor response (VMR) to different CRD pressures. The distribution and expression levels of PAR-2, CGRP and c-Fos in the spinal cord dorsal horn (SCDH) were detected to investigate the central regulating mechanism of EA for controlling the VH.

## Results

### TNBS-induced ileal inflammation

**Figure 1:**
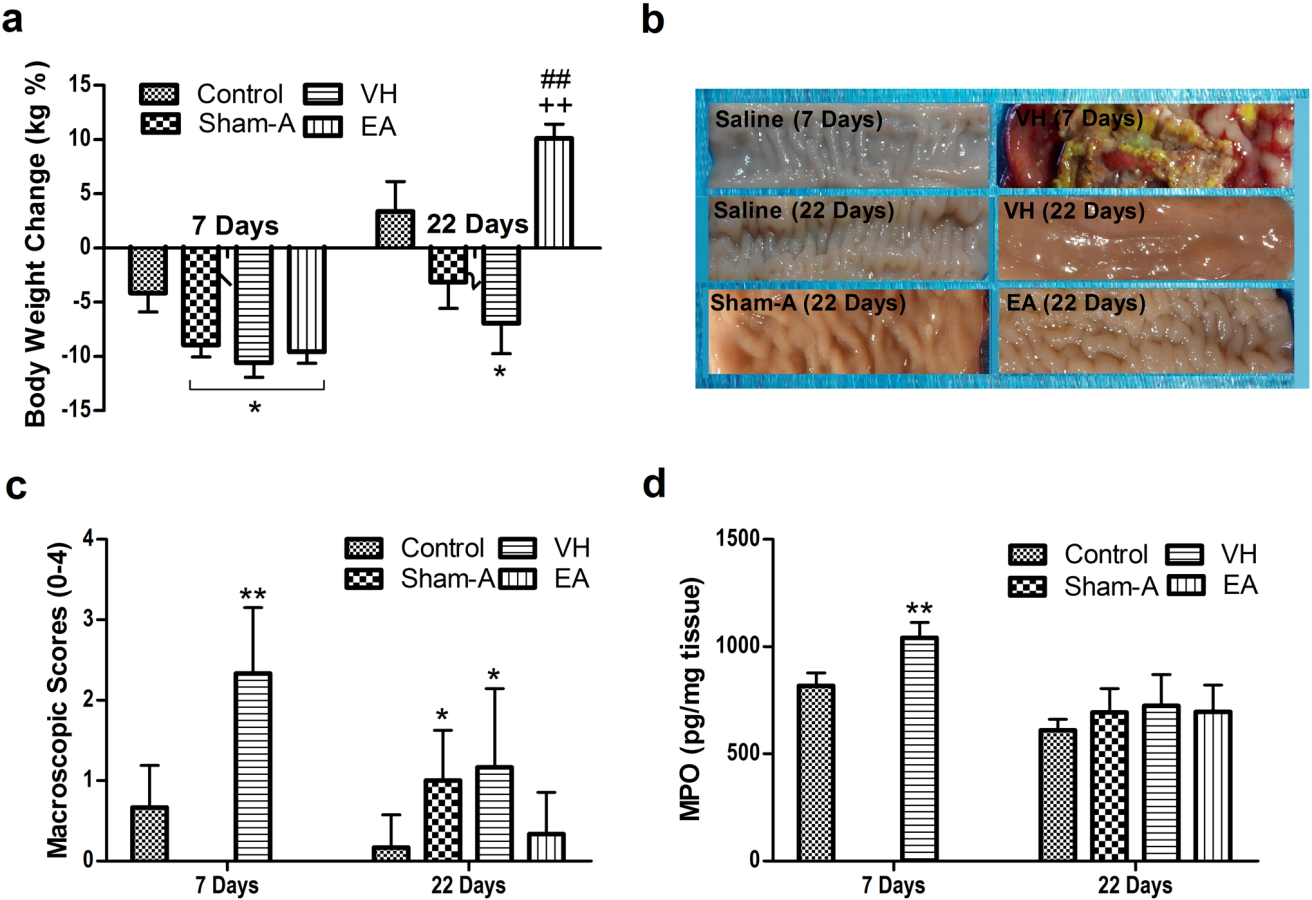
Effects of EA treatments on body weight, macroscopic pathologic changes and MPO concentrations in goats (mean ± SD). (**a**) Body-weight change. Body-weight changes were analyzed with one-way ANOVA [F (3, 32) = 5.380, *P* = 0.004] followed by Bonferroni’s post-hoc test (*P* = 0.008, VH group vs. Control group; n = 12; *P* = 0.045, Sham-A vs. Control group, *P* = 0.049, EA group vs. Control group; n = 6). The goats of VH, Sham-A and EA groups exhibited lower body weight at 7 days. The VH group showed lower body weight than control goats at 22 days. EA-group gained body weight than those of VH or Sham-A group at 22 days. (**b**) Ileal histopathological changes. Ileal segments were incised longitudinally for macroscopic observation and representative images were taken from NaCl- and TNBS-treated goats on day 7 and from all groups on day 22. TNBS-injected ileum revealed hemorrhage, necrosis, edema, adhesion with its loop or mesentery, ulcerations and thickened wall only on day 7. (**c**) Ileal macroscopic scores. Macroscopic change scores were scored by two independent observers employing a 0–4 scale. Macroscopic change scores increased [χ^2^(2) = 7.221, *P* = 0.007, df = 1 with a mean rank macroscopic scores of 12.75 for VH group, 3.83 for Control group] in the ileum of VH group on day 7 (*P* = 0.007, n = 6). Similarly, Sham-A (*P* = 0.026, n = 6) and VH-group (*P* = 0.05, n = 6) showed higher macroscopic scores as compared to Control group on day 22. Kruskal–Wallis analysis followed by Mann–Whitney U-test. (**d**) Ileal myeloperoxidase (MPO) concentrations (mean ± SD, n = 6). MPO concentrations were analyzed with one-way ANOVA [F (1, 10) = 34.896, *P* = 0.000] followed by Independent t test (*P* = 0.000). TNBS-injected ileal samples displayed markedly higher MPO concentrations only on day 7. **P  < 0.01; *P < 0.05, VH/Sham-A/EA group versus Control group; ^++^P < 0.01, VH group versus Sham-A group; ^##^P < 0.01, VH group versus EA group.

**Figure 2:**
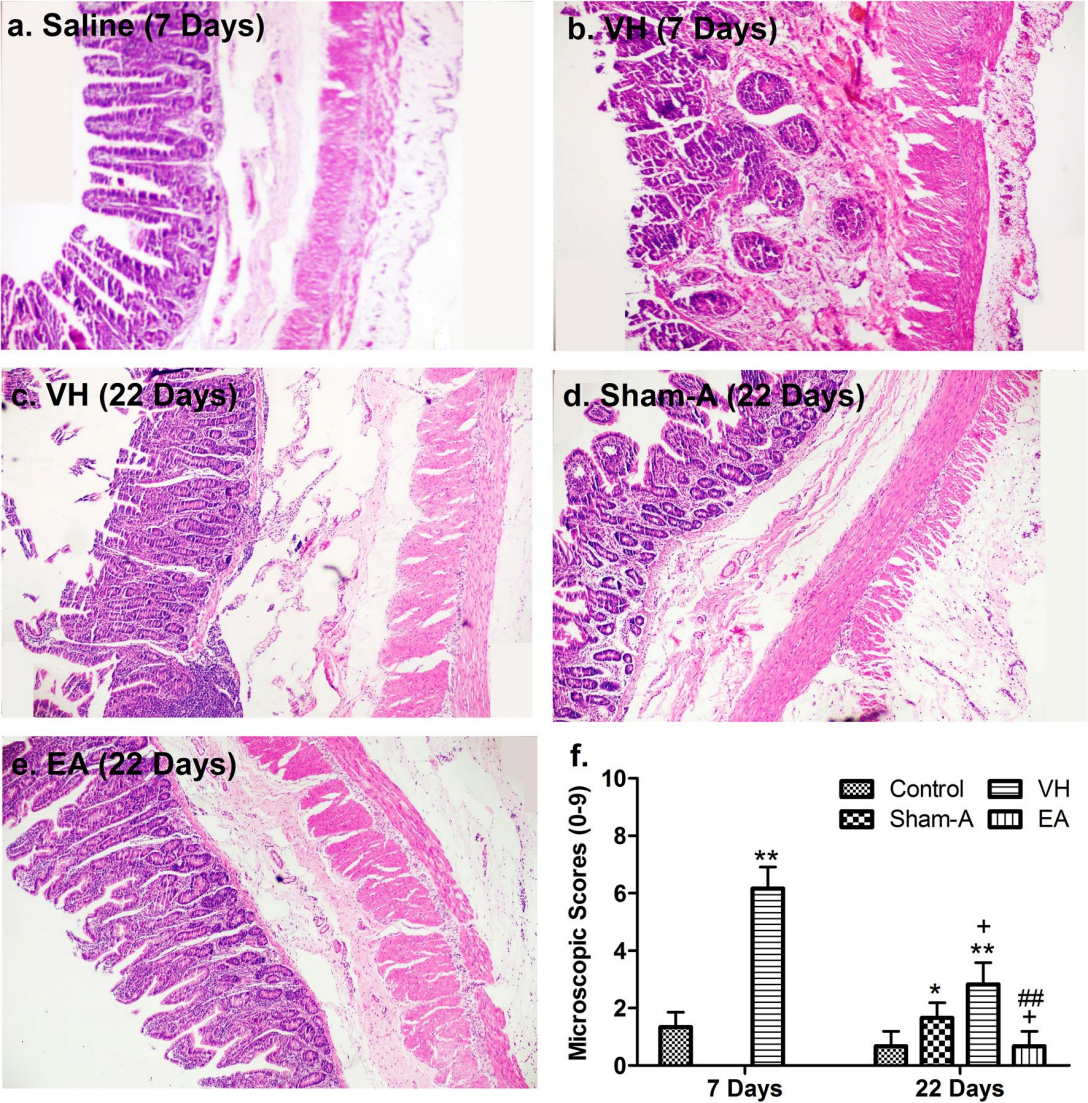
Effects of TNBS administration and EA stimulations on microscopic pathological changes in the ileum of goats (Mean ± SD, n = 6). Microscopic changes in the distal ileal section stained with hematoxylin and eosin (H&E) at days 7 and 22. Representative images (20×) were taken from Control and VH group on day 7 and from VH, Sham-A and EA groups on day 22 (**a**–**e**). The microscopic changes were assessed and scored by two pathologists unawared with the treatment status on a 0–9 scale, i.e., crypt architecture (0–3), inflammatory cells infiltration (0–3), and ulceration (0–3) (**f**). A Kruskal–Wallis H test showed a significant difference in microscopic scores between the Control and VH group, χ^2^(2) = 8.800, *P*  =  0.003, df  =  1 with a mean rank microscopic scores of 3.50 and 9.50, respectively at 7 days. A Mann–Whitney U-test indicated that the microscopic scores of VH group increased (*P*  =  0.003) significantly than Control group on day 7. Microscopic scores [χ^2^  =  17.436, *P*  =  0.001, df  =  3 with a mean rank microscopic scores of 7.17 for Control, 14.83 for Sham-A, 20.83 for VH and 7.17 for EA group] at 22 days. Microscopic scores were remarkably increased in the VH group (*P*  =  0.003) and Sham-A group (*P*  =  0.014) than the Control group. However, the EA group resulted in significantly lower microscopic scores than those of Sham-A (*P*  =  0.014) and VH group (*P*  =  0.003) on day 22. **P < 0.01; *P < 0.05, VH/Sham-A group versus Control group; ^+^*P* < 0.05, VH/EA group versus Sham-A group; ^##^*P* < 0.01, VH group versus EA group.

TNBS-administrated goats demonstrated mucoid or bloody diarrhea, sluggishness and gradual weight loss. As compared with Control group, the goats of VH, Sham-A and EA groups resulted in lower (*P* = 0.008, 0.045 and 0.049) body weight at 7 days [F (3, 32) = 5.380, *P* = 0.004]. The body weight did not differ (*P* > 0.05) among the VH, Sham-A and EA groups. The goats of VH group exhibited lower (*P* = 0.039) body weight than that of the Control at 22 days [F (3, 20) = 9.755, *P* = 0.0003]. The EA-treated goats showed greater (*P* = 0.0003 or 0.005) body weight than the goats of the VH or Sham-A group at 22 days (Fig. 1a). No obvious histopathological lesions were observed on saline-treated ilea at days 7 and 22. However, the TNBS-injected ilea showed hemorrhage, necrosis, edema, adhesion with intestinal loop or mesentery, ulcerations and thickened ileal wall on day 7 (Fig. 1b).The segments cranial (jejunum) and caudal (cecum and colon) to the TNBS-injected ileum did not reveal any pathological lesions. Microscopically, the TNBS-injected ilea exhibited disarranged intestinal villi, distorted epithelial crust, shorter crypt, fewer goblet cells, the increased neutrophils and lymphocytes, granulomatous lesions and discrete edema at days 7 and 22 (Fig. 2). However, the ilea of EA-treated goats revealed no histopathologically remarkable changes on day 22.

The ilea of VH group revealed remarkably higher (*P* < 0.01) macroscopic (U = 2.000, *P* = 0.007) and microscopic changes (U = 0.000, *P* = 0.003) scores than the control on day 7 (Figs. 1c, 2). As compared with control, the macroscopic (U = 7.500, *P* = 0.05 and U = 5.500, *P* = 0.026) and microscopic (U = 0.000, *P* = 0.003 and U = 4.000, *P* = 0.014) change scores in the ileum of VH and Sham-A groups were increased (*P* < 0.05, 0.01) on day 22. However, no difference (*P* > 0.05) in macroscopic and microscopic change scores was found between ileal samples of Control and EA groups. The ilea of Sham-A (U = 4.000, *P* = 0.014) and VH (U = 0.000, *P* = 0.003) groups exhibited an increased (*P* < 0.05, 0.01) change in microscopic scores as compared to the ilea of EA-treated goats. Likewise, the TNBS-injected ileal segments showed markedly higher (*P* < 0.01) MPO concentrations [F (3, 20) = 9.503, *P* = 0.000] than NaCl-injected ilea on day 7 (Fig. 1d). However, no changes (*P* > 0.05) in MPO concentrations among the four groups were found on day 22.

### Cumulative effects of repetitive EA intervention on VMR to CRD

**Figure 3:**
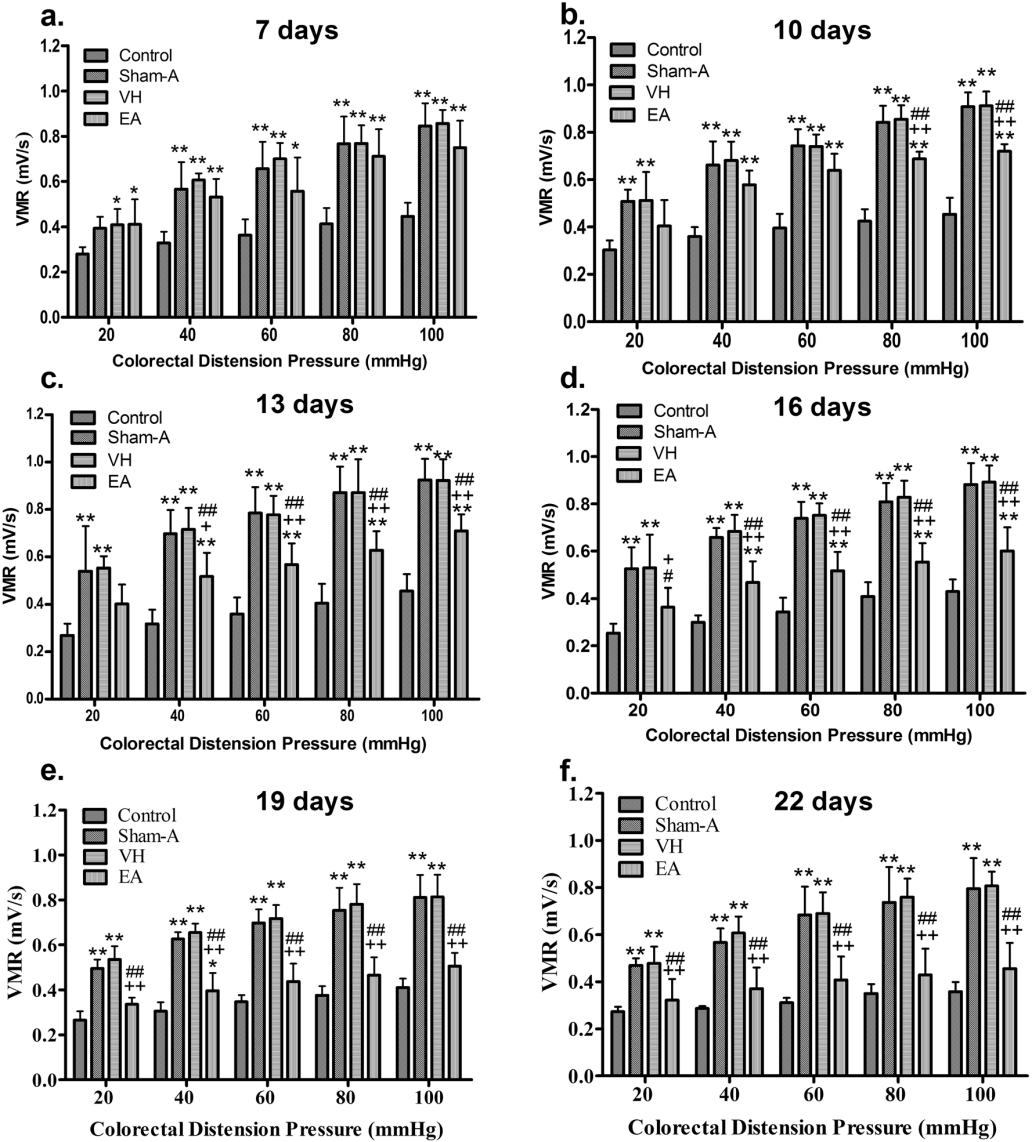
Effects of EA treatments on visceral hypersensitivity, measured by electromyography (EMG) of abdominal muscles’ visceromotor responses (VMR) to colorectal distension pressure (CRD) (Mean ± SD, n = 6). The EMG and mechanical irritation signals were quantified using the MedLab U/4C501 software (Nanjing Medease Science and Technology Co., Ltd, China, https://www.medease.com.cn). EMG activities during distensions minus resting activities were considered as VMRs to CRD, and are expressed as millivoltage per-second (mV/s). The VMRs were increased with higher CRD pressures (**a**–**f**). VH or EA group versus Control group (*P* < 0.05; ANOVA *P* = 0.016) on day 7; Sham-A or VH or EA group (*P* < 0.01, 0.05) versus Control group with 40–100 mmHg (ANOVA *P* = 0.000) at days 7–22; Sham-A or VH group (*P* < 0.01) versus Control group with 20 mmHg on day 10 (ANOVA *P* = 0.001) and 13–22 (ANOVA *P* = 0.000).; Sham-A or VH group (*P* < 0.01) versus EA group with 80–100 mmHg (ANOVA *P* = 0.000) on day 10; Sham-A or VH group versus EA group (*P* < 0.01, 0.05) with 40–100 mmHg (ANOVA *P* = 0.000) on day 13; Sham-A or VH group versus EA group (*P* < 0.01, 0.01) with 20–100 mmHg at days 16–22. **P < 0.01, *P < 0.05, VH/ Sham-A/EA group versus Control group. ^++^*P* < 0.01 and ^+^*P* < 0.05, Sham-A group versus EA group; ^##^*P* < 0.01, ^#^*P* < 0.05, VH group versus EA group. One-way ANOVA followed by Bonferroni’s post-hoc test.

The EMG was used to measure VMR to graded CRD pressures (20–100 mmHg). The VMR were intensified with increased strength of CRD pressures (Fig. 3). As compared with the control, VH and EA group exhibited higher (*P* = 0.039 or 0.035) VMR with 20 mmHg [F (3, 20) = 4.365, *P* = 0.016] on day 7. However, the goats of Sham-A or VH or EA group displayed remarkably higher (*P* < 0.01) VMR with 20 mmHg [F(3, 20) = 7.548, *P* = 0.001 on day 10, F(3, 20) = 9.473, *P* = 0.000 on day 13, F(3, 20) = 12.102, *P* = 0.000 on day 16, F(3, 20) = 49.825, *P* = 0.000 on day 19 and F(3, 20) = 19.969, *P* = 0.000 on day 22) and 40–100 mmHg [F(3, 20) = 15.828, 23.169, 27.221, 48.858, 67.556 and 34.565 for days 7, 10, 13, 16, 19 and 22, respectively; *P* = 0.000 with 40 mmHg; F(3, 20) = 11.224, 38.269, 30.568, 51.912, 52.756 and 26.582 for days 7, 10, 13, 16, 19 and 22, respectively; *P* = 0.000 with 60 mmHg; F(3, 20) = 16.717, 81.642, 26.191, 48.742, 37.975 and 24.473, respectively, *P* = 0.000 with 80 mmHg; F(3, 20) = 28.177, 78.592, 45.258, 46.739, 42.061 and 37.984 for days 7, 10, 13, 16, 19 and 22, respectively, *P* = 0.000 with 100 mmHg] at days 10–22 and days 7–22, respectively. The EA-treated goats revealed lower (*P* < 0.01) VMR with 80–100 mmHg [F(3,20) = 81.642 with 80 mmHg and 78.592 with 100 mmHg, *P* = 0.001] CRD pressures on day 10, with 40–100 mmHg CRD pressures [F(3,20) = 27.221 with 40 mmHg, 30.568 with 60 mmHg, 26.191 with 80 mmHg, 45.258 with 100 mmHg; *P* = 0.000] on day 13, and with all CRD pressures [F(3, 20) = 12.102, 49.825 and19.969 for days 16, 19 and 22, *P* = 0.000 with 20 mmHg; F(3,20) = 48.858, 67.556 and 34.565 for days 16, 19 and 22, *P* = 0.000 with 40 mmHg; F(3, 20) = 51.912, 52.756 and 26.582 for days 16, 19 and 22, *P* = 0.000 with 60 mmHg; F(3, 20) = 48.742, 37.975 and 24.473 for days 16, 19 and 22, *P* = 0.000 with 80 mmHg; 46.739, 42.061, 37.984 for days 16, 19 and 22, *P* = 0.000 with 100 mmHg) at days 16–22 as compared to the goats of Sham-A or VH group. Compared with the initial values, the repetitive EA applications attenuated VMR to 80 mmHg [F (6, 35) = 12.690, *P* = 0.000] and 100 mmHg [F (6, 35) = 17.432, *P* = 0.000] CRD pressures (*P* < 0.05, 0.01) on day 16. The magnitude of VMR to 40–100 mmHg [F (6, 35) = 7.613, *P* = 0.000 for days 19 and 22 with 40 mmHg; F(6, 35) = 6.738 for 22, *P* = 0.000 for days 19 and 22 with 60 mmHg; F(6, 35) = 12.690, *P* = 0.000 for days 16, 19 and 22 with 80 mmHg; F(6, 35) = 17.432, *P* = 0.000 for days 16, 19 and 22 with 100 mmHg] decreased (*P* < 0.01) further with repetitive EA applications at days 19 and 22 (Table 1). However, the sham-acupuncture did not change VMR during the experiment.

**Table 1:** Cumulative effects of repetitive EA and Sham-A treatments on VMRs to different CRD pressures (mean ± SD, n = 6).

Group	Days	Visceromotor responses (mV/s)				
		20 mmHg	40 mmHg	60 mmHg	80 mmHg	100 mmHg
EA	0	0.40 ± 0.07	0.60 ± 0.03	0.70 ± 0.07	0.76 ± 0.08	0.85 ± 0.06
	7	0.41 ± 0.11	0.53 ± 0.08	0.55 ± 0.15	0.71 ± 0.12	0.74 ± 0.12
	10	0.40 ± 0.11	0.57 ± 0.06	0.63 ± 0.07	0.68 ± 0.03	0.71 ± 0.03
	13	0.40 ± 0.08	0.51 ± 0.10	0.56 ± 0.09	0.62 ± 0.08	0.70 ± 0.07
	16	0.36 ± 0.08	0.46 ± 0.09	0.51 ± 0.08	0.55 ± 0.08**	0.60 ± 0.10**
	19	0.33 ± 0.03	0.39 ± 0.08**	0.43 ± 0.08**	0.46 ± 0.08**	0.50 ± 0.06**
	22	0.32 ± 0.09	0.37 ± 0.09**	0.40 ± 0.10**	0.43 ± 0.11**	0.45 ± 0.11**
Sham-A	0	0.40 ± 0.08	0.59 ± 0.03	0.67 ± 0.08	0.74 ± 0.10	0.83 ± 0.06
	7	0.39 ± 0.05	0.56 ± 0.12	0.65 ± 0.12	0.76 ± 0.12	0.84 ± 0.10
	10	0.50 ± 0.05	0.66 ± 0.10	0.74 ± 0.07	0.84 ± 0.07	0.90 ± 0.06
	13	0.53 ± 0.19	0.69 ± 0.10	0.78 ± 0.11	0.87 ± 0.11	0.92 ± 0.09
	16	0.52 ± 0.09	0.65 ± 0.04	0.73 ± 0.07	0.80 ± 0.08	0.88 ± 0.09
	19	0.49 ± 0.04	0.62 ± 0.03	0.69 ± 0.06	0.75 ± 0.10	0.81 ± 0.10
	22	0.46 ± 0.03	0.56 ± 0.06	0.68 ± 0.12	0.73 ± 0.15	0.79 ± 0.13

**P < 0.01, initial VMR values versus VMR values after EA stimulation in EA group. F(6, 35) = 7.613, *P* = 0.000 for days 19 and 22 with 40 mmHg; F(6, 35) = 6.738 for 22, *P* = 0.000 for days 19 and 22 with 60 mmHg; F(6, 35) = 12.690, *P* = 0.000 for days 16, 19 and 22 with 80 mmHg; F(6, 35) = 17.432, *P* = 0.000 for days 16, 19 and 22 with 100 mmHg. One-way ANOVA is followed by Bonferroni post-hoc test.

### Effects of repetitive EA on the distribution of spinal PAR-2, CGRP and c-Fos immunoreactivities

**Figure 4:**
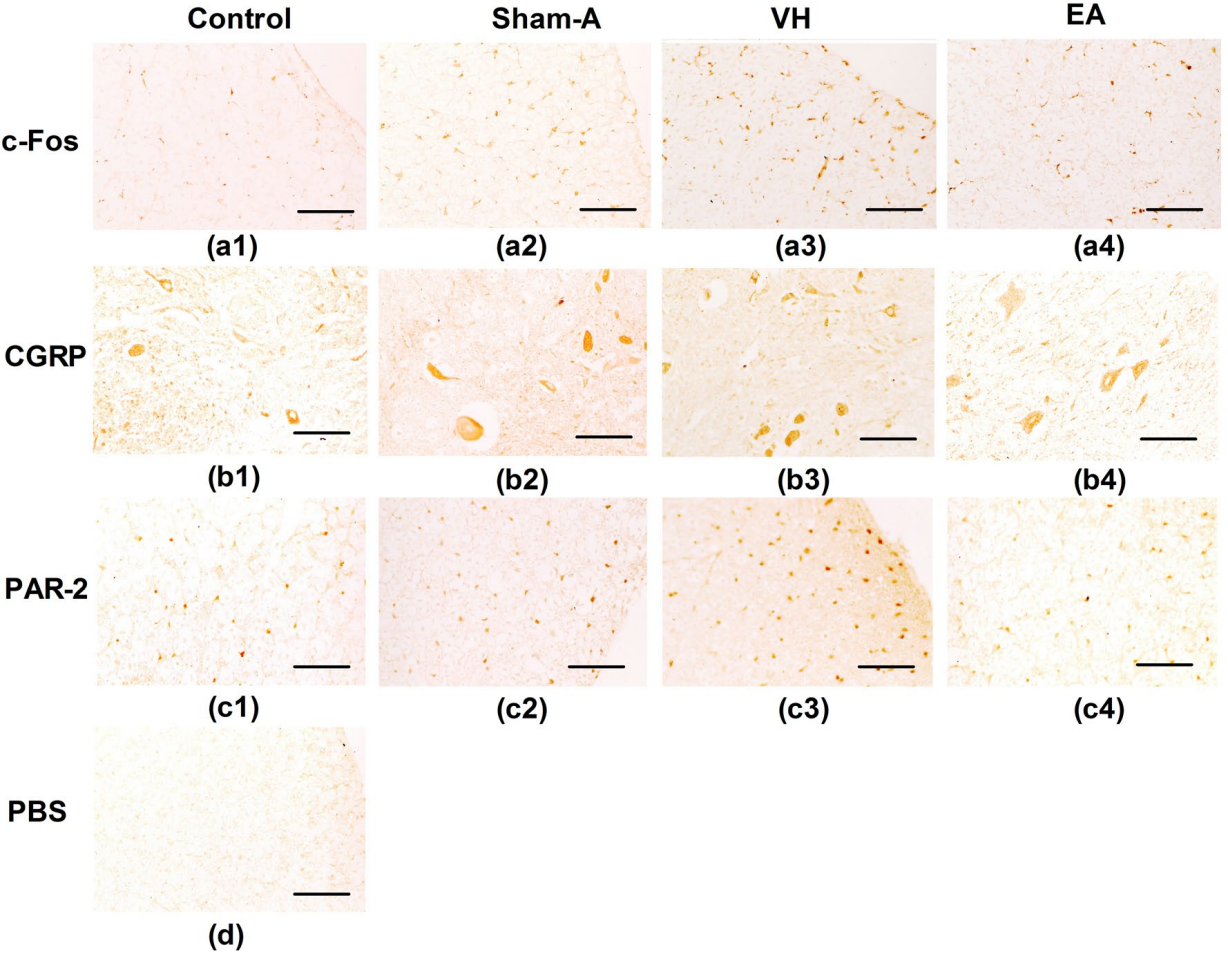
Distribution of PAR-2, CGRP and c-Fos proteins (20 ×) in the spinal cord of the goats on day 22 (n = 6). c-Fos positive neurons in the dorsal horn of spinal cord of Control (**a1**), Sham-A (**a2**), VH (**a3**) and EA group (**a4**). CGRP positive neurons (browns) in the dorsal horn of the spinal cord of Control (**b1**), Sham-A (**b2**), VH (**b3**) and EA group (**b4**). PAR-2 positive neurons (browns) in the dorsal horn of spinal cord of Control (**c1**), Sham-A (**c2**), VH (**c3**) and EA group (**c4**). Dorsal horn of spinal cord of PBS (d). Brown stain shows the nuclei of positive cells or cytoplasm. Scale bars = 50 µm.

The immunohistochemical staining of sections obtained from the spinal cord demonstrated variation in distributions of spinal PAR-2, CGRP and c-Fos immunoreactivities among four groups (Fig. 4). The goats of the VH group revealed an enhanced spinal PAR-2, CGRP and c-Fos immunoreactivities than the goats of Control, However, PAR-2, CGRP and c-Fos immunoreactivities were decreased in the spinal cord of EA-treated goats as compared to the goats of the VH group.

### Effects of repetitive EA on PAR-2, CGRP and c-Fos proteins

**Figure 5:**
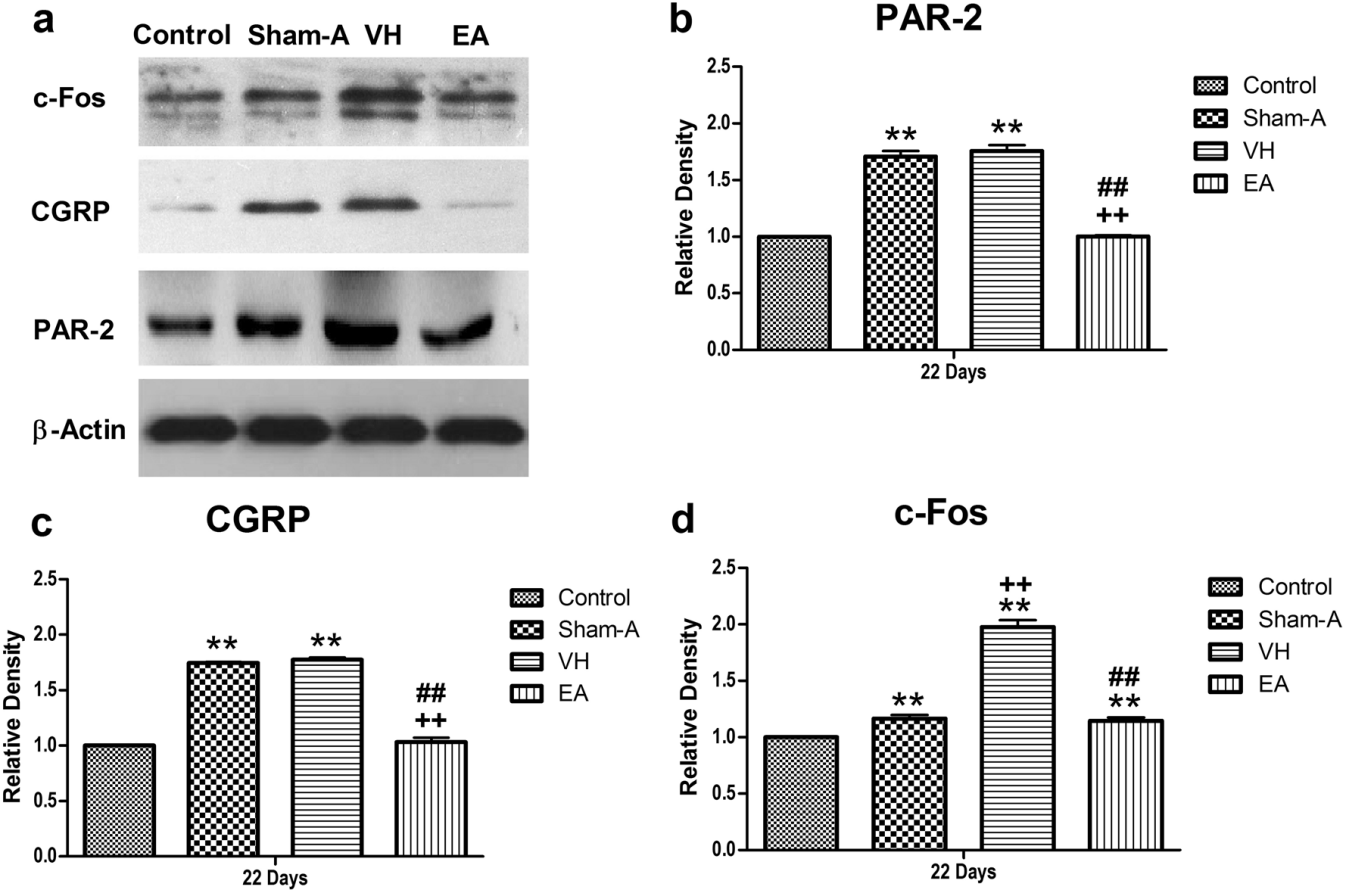
Cumulative effects of repetitive EA treatments on the expression of PAR-2, CGRP and c-Fos in the spinal cord of the goats. (a) Western blotting of PAR-2, CGRP and c-Fos in the spinal cord of the goats. All electrophoresis was performed under the same condition. The immuno-detected protein bands, appeared on a horseradish peroxidase substrate (Millipore, Billerica, MA, USA), were visualized using the ImageQuant LAS 4,000 min CCD camera (GE Healthcare, Piscataway Township, NJ, USA). The bands were analysed using a Quantity One software (Bio-Rad), and the result is expressed as the immunoreactivity ratio of the target gene to β-actin. Insets show a representative band of the proteins expressed in all groups. Full-length blots are presented in Supplementary Fig. S1. Data were normalized against β-actin and expressed as the mean ± SD, n = 6. Semi-quantitative analysis of (**b**) PAR-2 expressions; *P* = 0.000, VH/Sham-A group versus Control group; *P* = 0.000, VH group versus Sham-A group; *P* = 0.000, VH group versus EA group [F (3, 20) = 1,040.60, *P* = 0.000]. (**c**) CGRP expressions; *P* = 0.000, VH/Sham-A group versus Control group; *P* = 0.000, VH group versus Sham A group; *P* = 0.000, VH group versus EA group [F (3, 20) = 1819.47, *P* = 0.000]. (**d**) c-Fos expressions; *P* = 0.000, Sham-A or VH or EA group versus Control group; *P* = .000, VH group versus Sham-A group; *P* = 0.000, EA group versus VH group [F (3, 20) = 223.53, *P* = 0.000]. **P < 0.01, VH/Sham-A/EA group versus Control group; ^++^*P* < 0.01, VH/EA group versus Sham-A group; ^##^*P* < 0.01, VH group versus EA group. One-way ANOVA followed by Bonferroni’s post-hoc test.

The goats of Sham-A and VH groups showed remarkably higher (*P* = 0.00) spinal PAR-2 level [F (3, 20) = 1,040.60, *P* = 0.000] than the control. However, repetitive EA treatments resulted in slightly higher (*P* > 0.05) spinal PAR-2 level than the goats of Control, but remarkably lower (*P* < 0.01) spinal PAR-2 than VH or Sham-A group. Spinal CGRP [F (3, 20) = 1819.47, *P* = 0.000] changed in the same pattern as the PAR-2. The goats of VH or Sham-A or EA group exhibited an increased (*P* = 0.00) spinal c-Fos level [F (3, 20) = 223.53, *P* = 0.000] as compared with the control. Remarkably, the spinal c-Fos of EA-treated goats was higher (*P* < 0.01) than Control, but lower (*P* < 0.01) than that of VH-group (Fig. 5).

**Figure 6:**
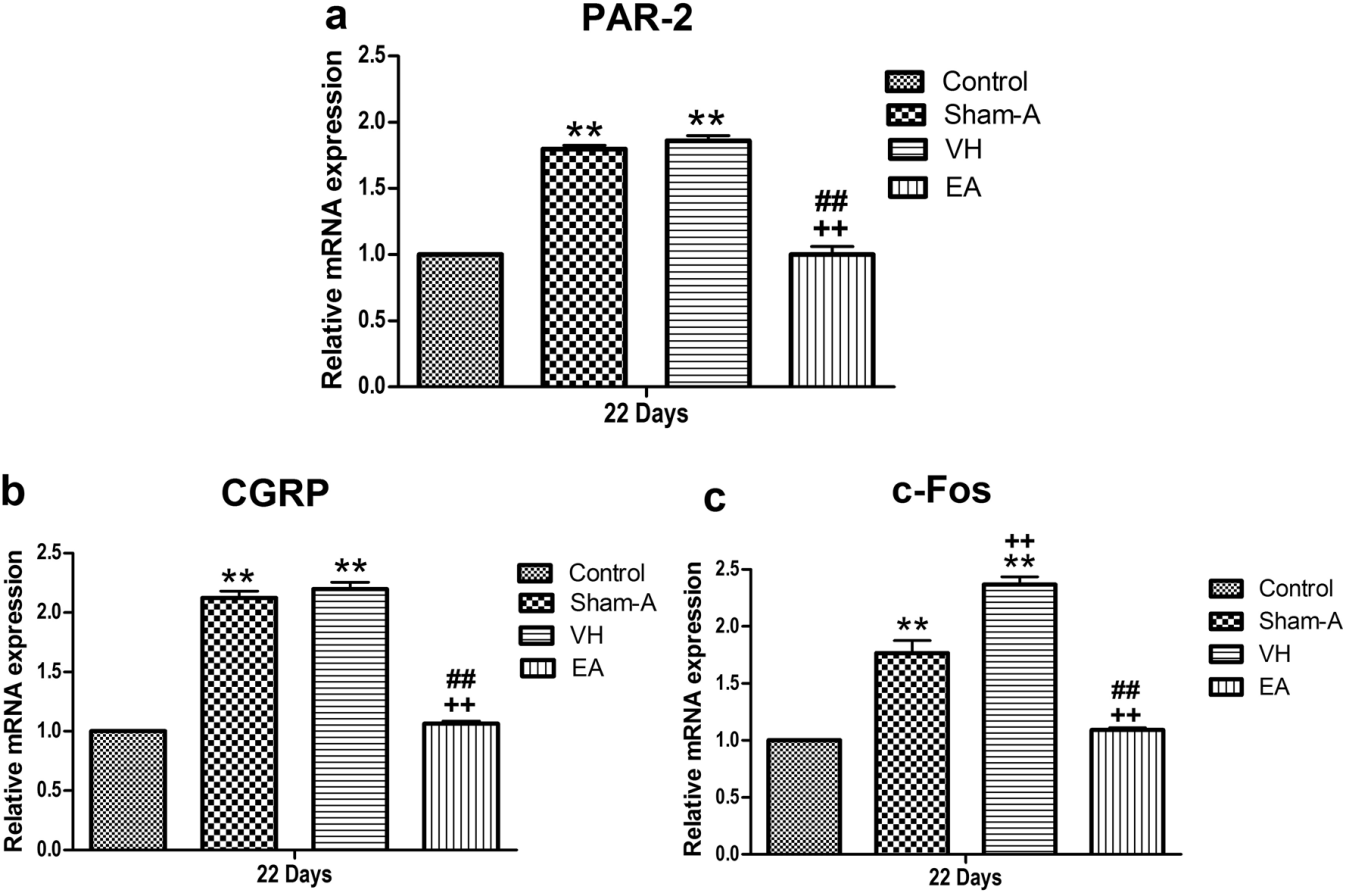
Cumulative effects of repetitive EA stimulations on the mRNA expressions of PAR-2 (a), CGRP (b) and c-Fos (c) in the spinal cord of the goats (Mean ± SD, n = 6). Quantitative real-time PCR (qRT-PCR) was performed to measure the mRNA **e**xpressions of PAR-2, CGRP and c-Fos (relative to GAPDH) in T_11_ thoracic spinal cord dorsal horns. (**a**) PAR-2 mRNA; *P* = 0.000, Sham-A or VH group versus Control group; *P* = 0.000, VH group versus Sham-A group; *P* = 0.000, EA group versus VH group [F (3, 20) = 675.15, *P* = 0.000]. (**b**) CGRP mRNA; *P* = 0.000, Sham-A or VH group versus Control group; *P* = 0.000, VH group versus Sham-A group; *P* = 0.000, EA group versus VH group [F (3, 20) = 1,437.07, *P* = 0.000]. (**c**) c-Fos mRNA; *P* = 0.000, Sham-A or VH group versus Control group; *P* = 0.000, VH group or EA group versus Sham-A group; *P* = 0.000, EA group versus VH group [F (3, 20) = 930.32, *P* = 0.000]. **P < 0.01, VH/Sham-A/EA group versus Control group. ^++^*P* < 0.01, VH/EA group versus Sham-A group. ^##^*P* < 0.01, VH group versus EA group. One-way ANOVA followed by Bonferroni’s post-hoc test.

The spinal mRNA of PAR-2 [F (3, 20) = 675.15, *P* = 0.000], CGRP [F (3, 20) = 1,437.07, *P* = 0.000] and c-Fos [F (3, 20) = 930.32, *P* = 0.000] in Sham-A, VH and EA groups changed in the same pattern as their protein levels. Sham-A, VH and EA groups showed higher mRNA levels of spinal PAR-2, CGRP and c-Fos compared with the Control. Spinal mRNA level of EA group was lower (*P* < 0.01) than those of VH or Sham-A group, but not different (*P* > 0.05) from that of the Control (Fig. 6).

## Discussion

Visceral hypersensitivity (VH), an important symptom of IBDs and IBS, has received great interest in the recent years. However, VH originated from the distal part of the ileum still remains unexplored. Several researchers used the colitis model for studying the VH, which is not suitable for investigating the exact underlying pathogenesis of ileitis-induced VH because of its different anatomical location, the severity of inflammation, gut microbiome as well as spinal afferent pathways. Shah et al.^40^ administered TNBS/ethanol solution into the rat’s ileal lumen to induce transmural ileitis including granuloma and VH, which lasted over 20 days. Besides rodents, other animals have also been used for studying the ileitis and VH^41–43^. Goats are the appropriate animals for investigating the mechanism by which EA attenuates VH because of their profound sensitivity to EA stimuli and easier handling with minimal stress. Tahir et al.^42^ injected TNBS/ethanol solution (1.2 ml; 30 mg TNBS in 40% ethanol) in goat’s ileal wall through laparotomy and showed ileal inflammation for 21 days and VH for at least 28 days. Recently, MPO activity has been assayed as a biochemical indicator of inflammation. It is observed in the neutrophils and their oxidative products, particularly involved in the tissue damage process during the acute stage of inflammation. In the present experiment, TNBS/ethanol (30 mg in 30% Ethanol) injection in the goat’s distal ileal wall resulted in remarkably increased MPO concentration, macroscopic and microscopic inflammatory change scores, and transmural ileitis on day 7. Feng et al.^44^ evaluated MPO activity in naive rats and TNBS-treated colon of rats at days 2, 7, 10, 14, 28 and 42, and found the increased MPO concentration till 14 days in TNBS-treated colon of rats. Our earlier study^40^ also showed that the normalization of MPO concentration in TNBS-treated ileal tissue occurred after 14 days. Likewise, Wan et al.^32^ reported that the MPO concentration in TNBS-injected ileum of the goats was increased on day 7 rather than on day 22. Increasing sets of evidence show the importance of PAR-2 both in gastroenteritis^11,45,46^ and inflammatory condition of the central nerve system^17,47,48^. PAR-2 has been expressed in two-thirds of sensory neurons, and 40% of PAR-2 expressing sensory neurons coexpress CGRP and substances-P^17^. Proteases such as trypsin, tryptase and cathepsin-s, activate PAR-2 and cause the release of neuropeptides from the primary spinal afferents to release their peripheral and central terminals. The peripherally released neuropeptides inflict neurogenic inflammation while their central releases result in hyperalgesia^5^. Interestingly, proteases that activate the neuronal PAR-2 have been derived from the blood and mast cells, or are recruited into the neurons and astrocytes^49–52^. Intracolonically infused PAR-2 agonist (SLIGRL-NH_2_) results in the persistent VH, with an elevated spinal c-Fos expression as an indicator of the neuronal activation^53^. The majority of neurons showed increased CGRP expression after peripheral inflammation, and mice missing CGRP expression in the CNS failed to generate inflammation-induced hyperalgesia^54^. In this study, goats with TNBS-induced ileitis showed remarkably higher VMR to CRD and increased expressions of PAR-2 and CGRP in the SCDH. These studies indicate that intestinal VH development that may be caused by PAR-2 activated release of CGRP in CNS. However, the molecular involvement of spinal PAR-2 in ileitis-induced VH is unknown. It is well known that the PAR-2 is involved in signaling mechanism to regulate activity as well as expression of ion channels. The activated PAR-2 increases TRPV1 currents and regulates the TRPV1-induced Ca^2+^ signal transduction in neuronal cells via a protein kinase-C (PKC) dependent manner. Furthermore, PAR-2 activation enhances the capsaicin-evoked CGRP release^5,18^, which has been proved to be remarkably involved in the nociception^55–57^.

The c-Fos, a proto-oncogene protein, has been exclusively considered as a biological indicator of the activated central neurons or areas^34,35^. It can be rapidly expressed in the SCDH neurons after noxious stimulation. An earlier study^36^ reported the increased expression level of c-Fos in laminae-I and -II or -V and -VI of the SCDH after nociceptive stimulation. It plays a crucial role in the development of a pain state as part of spinal cord adaptive response to both constant and subsequent nociceptive input. However, EA stimulation activates c-Fos-immunoreactive cells on the laminae-III and -IV of the SCDH^34,58^, which is somewhat different from those caused by noxious stimulation. It is important to note that both high and low frequencies of EA stimulations suppress nociception-induced spinal c-Fos expression^59^. Qi and Li^60^ reported that EA significantly reduced chronic visceral hyperalgesia and provoked c-Fos expressions in SCDH superficial laminae. Therefore, c-Fos levels are commonly used to evaluate how EA modifies the neuronal regulatory mechanism underlying the neuropathic and inflammatory pain. In this study, c-Fos levels in the superficial laminae of the SCDH were increased during VH and ileitis, but decreased after EA treatment, indicating that intestinal inflammation- or VH-activated SCDH was the important site on which EA regulates. Accumulating sets of evidence supports the presence of VH due to cross-sensitization between the different internal organs in human^61–63^ and experimental animals^64–67^. Furthermore, Pan et al.^68^ described the sensitization of pelvic organs in rats with TNBS-induced colitis because colitis triggers the release of SP and CGRP in the urinary bladder. Likewise, CD pediatric patients coexisting functional GI disorders showed rectal hypersensitivity^2^. Our earlier studies^32,40,42^ also reported VH in response to graded CRD in rats and goats with TNBS-induced ileitis. Researches demonstrate that VH across the organs is due to the central sensitization of spinal cord neurons receiving convergent input from different organs^69,70^ or descending bulbospinal inhibition of sacral dorsal horn neurons in response to chronic intestinal tissue irritation^71^. Therefore, assessment of VMRs to CRD in TNBS-induced ileitis goats in our experiment is based on the rationale of central sensitization of spinal cord neurons receiving convergent input from both thoracolumbar and lumbosacral spinal afferents pathways.

EA has been used for relieving various pains^72–75^, and Zusanli points (ST36) are classic acupoints for treating the visceral pain. Wu et al.,^76^ demonstrated that EA stimulation at ST36 resulted in attenuation of the acute inflammation-induced visceral pain in rats. Furthermore, EA at ST36 relieved the visceral sensitivity and the gastrointestinal motility disorder in rats of IBS model^77^. Recently, Wan et al.^32^ reported that EA at ST36 reduced pain behavioral manifestations and VH in goats. In this study, EA application at bilateral ST36 reduced the TNBS-induced ileitis and VMR to CRD of goats, which is similar to the findings of the earlier study^32^. These experiments clearly demonstrated that EA has the potential for attenuating VH. However, its underlying mechanisms need to be further explored. Hu et al.^78^ reported EA at ST36 and Kulun (BL60) that attenuated inflammation-induced hyperalgesia and down-regulated the PAR-2 levels in dorsal root ganglion neurons. Sun et al.^33^ described that EA attenuates VH by suppressing the spinal CGRP in diarrhea-predominant IBS rats. In this study, TNBS injection induced a remarkable VH, and higher expressions of PAR-2 and CGRP in the SCDH of goats, which were reversed by repeated EA. Our study suggests that EA intervention of VH is involved in its downregulation of spinal PAR-2 and CGRP.

## Methods

### Animal grouping

**Figure 7:**
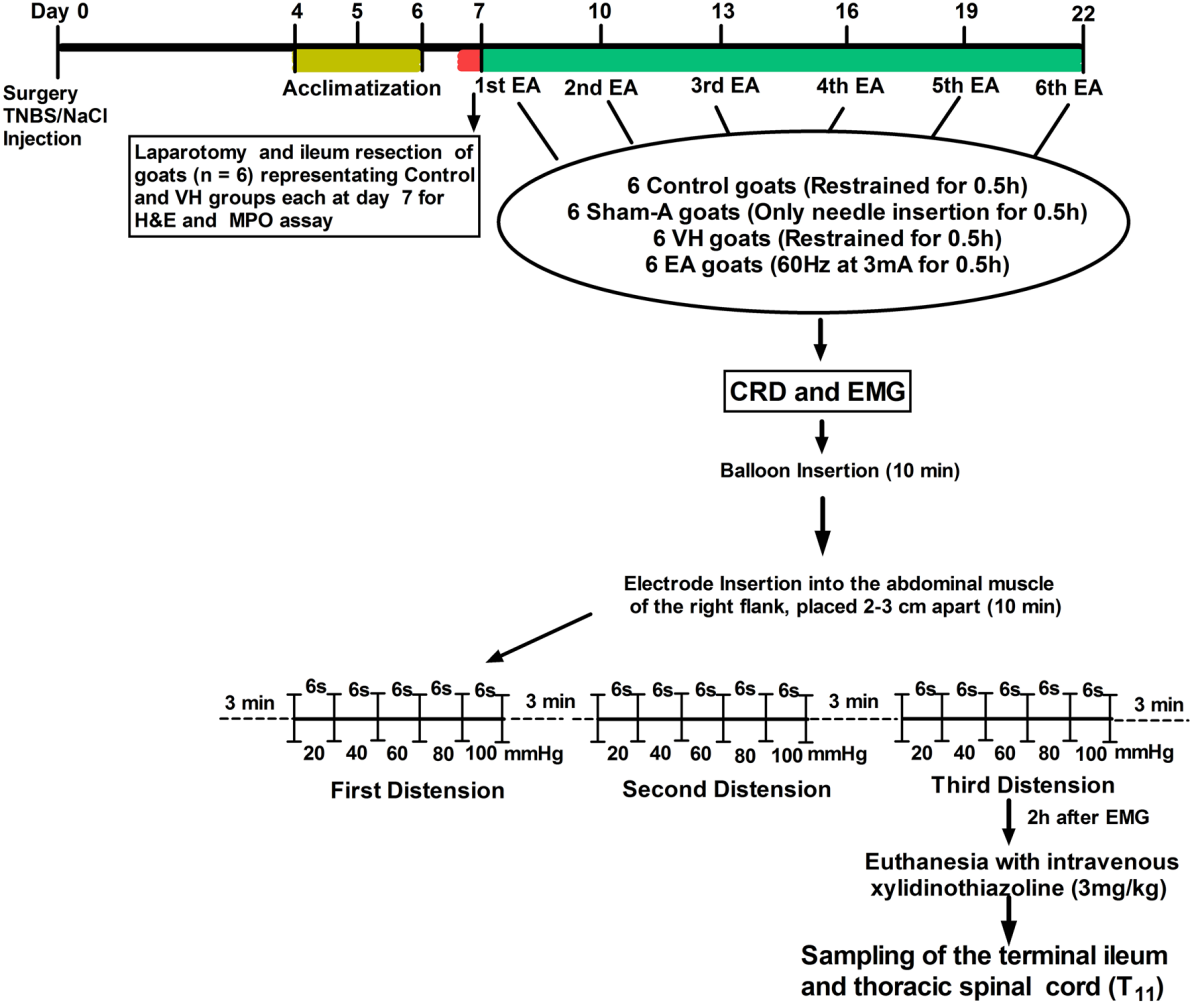
The scheme of electroacupuncture intervention experiment. All 36 goats of both sexes were randomly allocated in Control (n = 12), Sham-A (n = 6), VH (n = 12) and EA group (n = 6). Goats were anaesthetized and laparotomized for ileal injection of TNBS (1.2 ml, 30 mg in 30% ethanol) in Sham-A, VH and EA group and an equal volume of 0.9% NaCl in Control group at day 0. Goats were acclimatized for the working environment on postoperative days 4, 5 and 6. Six goats representing each VH and Control group were laparotomized on day 7 for the resection of terminal ileum segments for H&E and MPO assay. Goats of all groups were restrained on sternal position. In goats of EA group, electroacupuncture (60 Hz, 3 mA) was used to stimulate the bilateral ST-36 acupoints for 0.5 h firstly at 7 days and thereafter repeated every 3 days for 6 times. Sham-acupuncture was conducted for 0.5 h by inserting needles at the acupoints. A balloon catheter was inserted colorectally, and its tubular end was attached with a Y-connector to a vacuum-pump and sphygmomanometer for measurement of distension pressure. Two nickel-steel needles were inserted 2–3 cm apart as electrodes into the left abdominal musculature. The electrodes were fastened on the skin individually and connected to an EMG-apparatus. VMRs to CRD pressures were recorded by EMG. The balloon was distended for 6 s with 20 mmHg and pressures were increased continuously in a ramp mode to 40, 60, 80 and 100 mmHg at an interval of 6 s. CRD and EMG were recorded three times at an interval of 3 min. Immediately, goats were euthanized to collect the terminal-ileum for evaluation of ileitis and spinal cord (T_11_) for distribution and expression-levels of PAR-2, CGRP and c-Fos on day 22.

A total of 36 goats (20 males and 16 females with the dry period, i.e., not pregnant); 1-year-old and weighing 24.28 ± 5.01 kg were bought from Hubei Agricultural Academy of Science. Goats were fed on dried grass supplemented with a concentrated ration, and availed drinking water ad libitum. The clinically healthy goats were acclimatized to the surroundings for a period of 15 days. The experiments involving animals were performed according to the stipulated rules for experimental usage of laboratory animals (the regulation of the administration of affairs concerning experimental animals of P.R. China). All protocols were approved by the Laboratory Animal Research Center, Hubei and the ethical committee of Huazhong Agricultural University (Permit number: HZAUMO-2015–12). The goats were randomly allocated into the Control (n = 12), Sham-Acupuncture (Sham-A; n = 6), Visceral hypersensitivity (VH; n = 12), and Electroacupuncture (EA; n = 6). All experimental goats were injected with a solution of TNBS/ethanol and 0.9% NaCl into the ileal wall through laparotomy to induce VH on day 0. The 6 goats taken from each of the VH group (4 male and 2 female) and Control group (2 male and 4 female) on day 7 were then laparotomized to transect the terminal ileal segments for assessment of ileal inflammation while the reamined 24 goats (3 male and 3 female in each group) were euthanized to sample the terminal ileum for evaluating ileitis, and the spinal cord for distribution and expression levels of PAR-2, CGRP and c-Fos on day 22. The scheme of EA intervention has been demonstrated in Fig. 7.

### Induction of visceral hypersensitivity

All experimental goats were made to fast for 24 h to avoid regurgitation and respiration complications before the experiments began. After clinical examination, the goats were pre-medicated with atropine sulphate (0.02 mg/kg IM; Tianjin Pharmaceutical Group Xinzheng Co., Ltd, China) and Xylazole-HCl (0.15 mg/kg IV; North China Pharmaceutical Co Ltd, Shijiazhuang, China) and the inverted L-block was achieved with local infiltration of 2% Lidocaine-HCl (Shandong Hualu Pharmaceutical Co. Ltd., China) dorsally and cranially to the mid right flank. The endotracheal intubation was performed in all the goats to prevent the aspiration of ruminal ingesta. The right flank laparotomy was performed to exteriorize the terminal ileum on moist sterile gauze. For the goats in Sham-A, VH and EA groups, 1.2 ml (30 mg TNBS; Sigma Aldrich, USA, mixed in 30% Ethanol) was injected at five points on the non-mesenteric side of ileum, just 15 cm anteriorly from the ileo-cecal junction, with a 30-G needle attached to a 2-CC syringe. The TNBS dose was ascertained based on our earlier experiment^79^. For goats in the Control group, an equal volume of 0.9% NaCl was injected into the ileal wall following the aforementioned method. The cranial and caudal margin of the injection area was marked with two loosely placed silk ligatures (3–0) in the mesentery for easy identification during sampling time. The abdominal incision was closed as usual. Once recovered from anaesthesia, the goats were fed on dried grass supplemented with concentrated ration and availed free access to drinking water. The goats were monitored daily, and their incised wounds were dressed with 1% povidone-iodine twice a day daily till healing. Goats were injected with Tramadol-HCl (4 mg/kg, IM; Hubei Qianjiang Pharmaceutical Co., Ltd, China) up to 3 days for the relief of post-surgical pain. For the prevention of infection, Ampicillin (10 mg/kg, IM; Wuhan Shu Ou Technology Co., Ltd, China) was administered twice a day for upto 5 days.

### Assessment of ileal inflammation

The body weight, feed and water consumption and mortality rate of the experimental goats were recorded appropriately, while the fecal consistency and hemorrhage in feces were evaluated at days 0, 3 and 7. After overnight fasting, 6 goats from each of the VH and Control groups on day 7 underwent the same laparotomy procedure as described above for partial ileal samples for macroscopic and microscopic assessment and MPO assay. In brief, the mesenteric arteries and veins of the intestine to be resected were properly ligated with catgut, and the intestinal content was milked away. Two intestinal forceps were used to occlude the cranial and caudal ends of the intestinal segment. Approximately, a 5 cm ileum was resected from the previously marked area. After being washed in PBS solution, the intestinal segment was longitudinally incised and spread out on a sterilized drape to score the macroscopic lesions by two independent observers employing a 0–4 scale modifying the earlier criteria^79^. An anterior 2 × 2 cm intestinal segment was cut from the non-mesenteric side and fixed in a 4% buffered formaldehyde solution for the observation of microscopic lesions. A distal 2 × 2 cm non-mesenteric intestinal segment was weighed, frozen in liquid nitrogen (N_2_) and stored at − 80 °C for measuring the MPO concentration. Parallelly, an experienced surgical team anastomosed the two intestinal segments together in an end-to-end pattern, and laparotomy incision was closed in a routine manner. Goats were kept in a quiet room and observed till completely recovered from anaesthesia. Postoperative care was performed as mentioned above. The goats were sent to farms after 2 weeks of recovery to resume their normal lives.

The ileal tissue sections were performed following our previous report^40^. Briefly, the formaldehyde-fixed ileal specimens (2 × 2 cm) were embedded in paraffin, microtomed at 5 μm and mounted properly on polylysine coated slides. The three sequential slides were stained with H & E for observation (20 ×) with a microscope (Nikon Eclipse 80I, Nikon Corporation, Tokyo, Japan). The microscopic lesions were assessed and scored by two pathologists who were unaware of the experimental protocol using a 0–9 scale as described previously^80^. The distal ileal specimens were ground, homogenized at 4 °C in 1 ml PBS (pH 7.2) and centrifuged at 5,000 g at 4 °C for 10 min. The aliquots were separated to measure their protein concentrations using a Nanodrop Spectrophotometer (Thermo Fisher Scientific, Inc., USA). The MPO concentration was evaluated in triplicate utilizing its specific ELISA Kit (eBioscience, Inc., San Diego, CA 92,121, USA). The MPO values are expressed as pg/mg.

### Electroacupuncture

A set of bilateral Hou-San-Li (i.e. equivalent to Zu-San-Li; ST-36 in humans) points described in veterinary medicine was chosen for EA^81^. Seven days after the surgery, a pair of sterile acupuncture needles (0.45 mm D × 7.5 cm L, Suzhou Medical Supplies, Co. Ltd., China) was inserted 2 cm deeper bilaterally at the Hou-San-Li points in hind legs of the goats of EA group. Here, we just inserted needles into the muscles but not implanted them surgically. This technique did not irritate the goats, and thereby VMR values were not interfered by them. The both bilaterally inserted needles were linked with the wires to the output terminal of an EA-machine (WQ-6F Electronic Acupunctoscope; Beijing Xindonghua Electronic Instrument Co., Ltd., Beijing, China). In this study, we used 60 Hz stimulation frequency because of its profound analgesic effect in goats^82^. After restraining the experimental goats in sternal recumbency, EA was applied at Hou-San-Li acupoints with a stimulation frequency of 60 Hz for 0.5 h. The intensity of stimulation was accustomed to a value that only induced mild muscular spasm at the acupoints and it was restricted below 3 mA so as to avoid the discomfort. In the Sham-A group, acupuncture needles were inserted at the same acupoints of the goats and kept for 0.5 h without any hand manipulation and electrical stimulation. The goats in Control and VH groups were restrained only for 0.5 h similarly as the EA-treated goats. The EA treatments were applied firstly on day 7 and thereafter repeated at an interval of 3 days, upto 6 times as reported earlier^32^.

### Visceromotor response to colorectal distension

Electromyographies (EMGs) were performed for the quantitative measurement of visceromotor responses at different CRD pressures. It was recorded in all the goats immediately after EA treatment from 6 a.m. to 11 a.m. at days 7, 10, 13, 16, 19 and 22 following the previously described methods^40,83^. Initially, the goats were accustomed to the testing room for a period of 3 days. A balloon catheter (8 cm long), made from the finger of a latex surgical glove, was lubricated with paraffin jelly. It was introduced 10 cm colorectally via the anus and its tubular end was attached using the Y-tubing to a vacuum-pump and sphygmomanometer for simultaneous distention of balloon and measurement of pressure. A pair of nickel-steel needles (0.45 mm D × 5 cm L) was introduced at a distance of 2–3 cm into the abdominal musculature of the left flank as electrodes. Thereafter, both electrodes were fastened to the skin and linked with a cable to an EMG-apparatus (Nanjing Medease Science and Technology Co. Ltd., China). The balloon was distended for 6 s with 20 mmHg and then to 40, 60, 80 and 100 mmHg in a continuous ramp mode at an interval of 6 s. Simultaneously, EMG was recorded for 6 s at each stage and then a 3 min break was done before another set of distention for a total of 3 sets (Fig. 7). The EMG and mechanical irritation signals were quantified using the software (MedLab-U/4C501, Nanjing Medease Science and Technology Co., Ltd, China). The differences of total areas of EMG during distensions and resting periods were considered as VMRs to CRD, and are expressed as millivoltage per-second (mV/s).

### Sample collection at the end of the experiment

Immediately after EMG, the xylidinothiazoline (3 mg/kg) was administered intravenously for euthanasia of the goats on day 22. Previously marked intestinal segment (5 cm) was excised and processed for assessing the inflammatory status with H & E and MPO concentration assay (as mentioned above in the assessment of ileal inflammation). The upper part of the eleventh thoracic spinal cord (T_11_) was excised and fixed in 4% buffered formaldehyde for IHC. While the lower part of the same spinal cord was frozen in liquid N_2_ and kept at − 80 °C for mRNA and protein extraction to be used for qRT-PCR and WB analysis, respectively.

### Immunohistochemistry

The fixed spinal specimens were processed, embedded properly in paraffin, sectioned using a microtome (5 μm), and mounted on glass slides coated with polylysine. Four sequential slides were processed for the SAB-immunohistochemistry procedure^32^. Briefly, these slides were incubated with mouse anti-PAR-2 antibody (Santa Cruz Biotechnology, CA, USA; 1:100 diluted in PBS), rabbit anti-CGRP antibody (Abcam Inc., Cambridge, MA-02139-1517, USA; 1:100 diluted in PBS), rabbit anti-c-Fos antibody (Santa Cruz Biotechnology, CA, USA; 1:200 diluted in PBS) and PBS (the negative control) for 12 h at 4 °C, respectively. Thereafter, the slides were incubated with their specific secondary antibodies (SAP 102-anti-mouse IgG Kit and SA1022-anti-rabbit IgG Kit, Boster Biological Technology Ltd., Wuhan, China) for 1 h at normal laboratory temperature. Finally, the slides were observed under the microscope using DAB for 2–3 min at normal laboratory temperature.

### Western blot analysis

The lower part of the T_11_ spinal cord was weighed and triturated in liquid N_2,_ and its protein was extracted using the RIPA-lysis buffer (Beyotime Biotech, Nantong, China). Protein concentration was quantified using a Nanodrop Spectrophotometer (Thermo Fisher Scientific, Inc., USA). Twenty micrograms of protein were loaded in each lane and differentiated by 10% or 12% SDS-PAG gel based on their molecular weight. The protein was transferred to PVDF-membranes and blocked in 3% BSA solution for 2 h min at normal room temperature. The blocked membranes were incubated using their primary antibodies, mouse anti-PAR-2 (1:500 in PBS; Santa Cruz Biotechnology, CA, USA), rabbit anti-CGRP (1:1,000 in PBS, Abcam Inc., Cambridge, MA-02139–1517, USA), rabbit anti-c-Fos (1:500 in PBS, Santa Cruz Biotechnology, CA, USA) and rabbit anti-β-actin (1:300, Santa Cruz Biotechnology, CA, USA), respectively, overnight at 4 °C. The membranes were washed and further treated with horseradish peroxidase conjugated secondary antibodies (goat anti-mouse-IgG/goat anti-rabbit IgG, 1:3,000 in PBS) for 1 h at normal room temperature. Visualization of the antigen–antibody complex was conducted with a horseradish peroxidase substrate (Millipore, Billerica, MA, USA) using the ImageQuant LAS 4,000 min CCD camera (GE Healthcare, Piscataway Township, NJ, USA). The bands were analysed using a Quantity One software (Bio-Rad), and the result is expressed as the immunoreactivity ratio of the target gene to β-actin.

### Real-time quantitative PCR

Total RNA was extracted from the spinal segment by the use of RNAprep pure cell kit (Tiangen, Beijing). The qRT-PCR was conducted using SYBR-Green (SYBR Green Real time PCR Master Mix QPK-201; Toyobo Co.) in One Step Plus™ real-time PCR system (Applied BioSystems, CA, USA). The primers sequences were used as the following: CTTCACGCCCAGTAACCT (PAR-F) and GATACCTGCATCCGCTTT (PAR-R); TCCCAGTTCATCCATCTCTACC (CGRP-F) and TGCAAATACAGCCACTACAACA (CGRP-R); CAGTGCCAACTTCATCCCA (c-Fos-F) and CAGCCATCTTATTCCTTTCC (c-Fos-R); TCTTCACTACCATGGAGAAGG-3′ (GAPDH-F) and TCATGGATGACCTTGGCCAG (GAPDH-R). The relative mRNA expressions of PAR-2, CGRP and c-Fos to GAPDH were determined following the previously used formula: 2^−∆∆CT^^84^.

### Statistical analysis

Data are represented as a mean ± SD. Data were analysed utilizing IBM-SPSS versus 23 (Armonk, NY: IBM Corp). Groups of comparisons were made using ANOVA, followed by Independent *t* test and Bonferroni post-hoc-test. The statistical differences in macroscopic and microscopic scores among groups were identified using Kruskal–Wallis analysis of variance followed by the rank-based Mann–Whitney U-test. A *P* < 0.05 was accepted as statistically significant.

## Supplementary information

Supplementary information

## Data Availability

All the data generated during this study are statically analysed and illustrated as table and figures. The dataset could be available on request from the corresponding author.
