# Supplementary material for: Electroacupuncture intervention of visceral hypersensitivity is involved in PAR-2-activation and CGRP-release in the spinal cord
Source: Sci Rep. 2020 Jul 7;10:11188. doi: 10.1038/s41598-020-67702-2 (PMC7341736; doi:10.1038/s41598-020-67702-2)
Supplement: Supplementary file 1 — Supplementary information [file 41598_2020_67702_MOESM1_ESM.docx]

**Supplementary Information**

**Electroacupuncture intervention of visceral hypersensitivity is involved in PAR-2-activation and CGRP-release in the spinal cord**

Manoj K Shah^1,2^, Yi Ding^1^, Juan Wan^1^, Habibullah Janyaro^1^, Adnan Hassan Tahir^1^, Vitaly Vodyanoy^3^_,_ Ming-Xing Ding^1*^

**Authors’ Affiliation**

1. College of Veterinary Medicine, Huazhong Agricultural University, Wuhan 430070, People’s Republic of China
2. Department of Surgery and Pharmacology, Agriculture and Forestry University, Nepal
3. Department of Anatomy, Physiology and Pharmacology, Auburn University, Auburn, AL, United States

***Corresponding author:**

**Ming-Xing Ding**

College of Veterinary Medicine

Huazhong Agricultural University

1 Shizishan Street, Hongshan District, Wuhan, Hubei Province, 430070

People’s Republic of China

Tel: +86 13808697687

Email: [dmx@mail.hzau.edu.cn](mailto:dmx@mail.hzau.edu.cn)


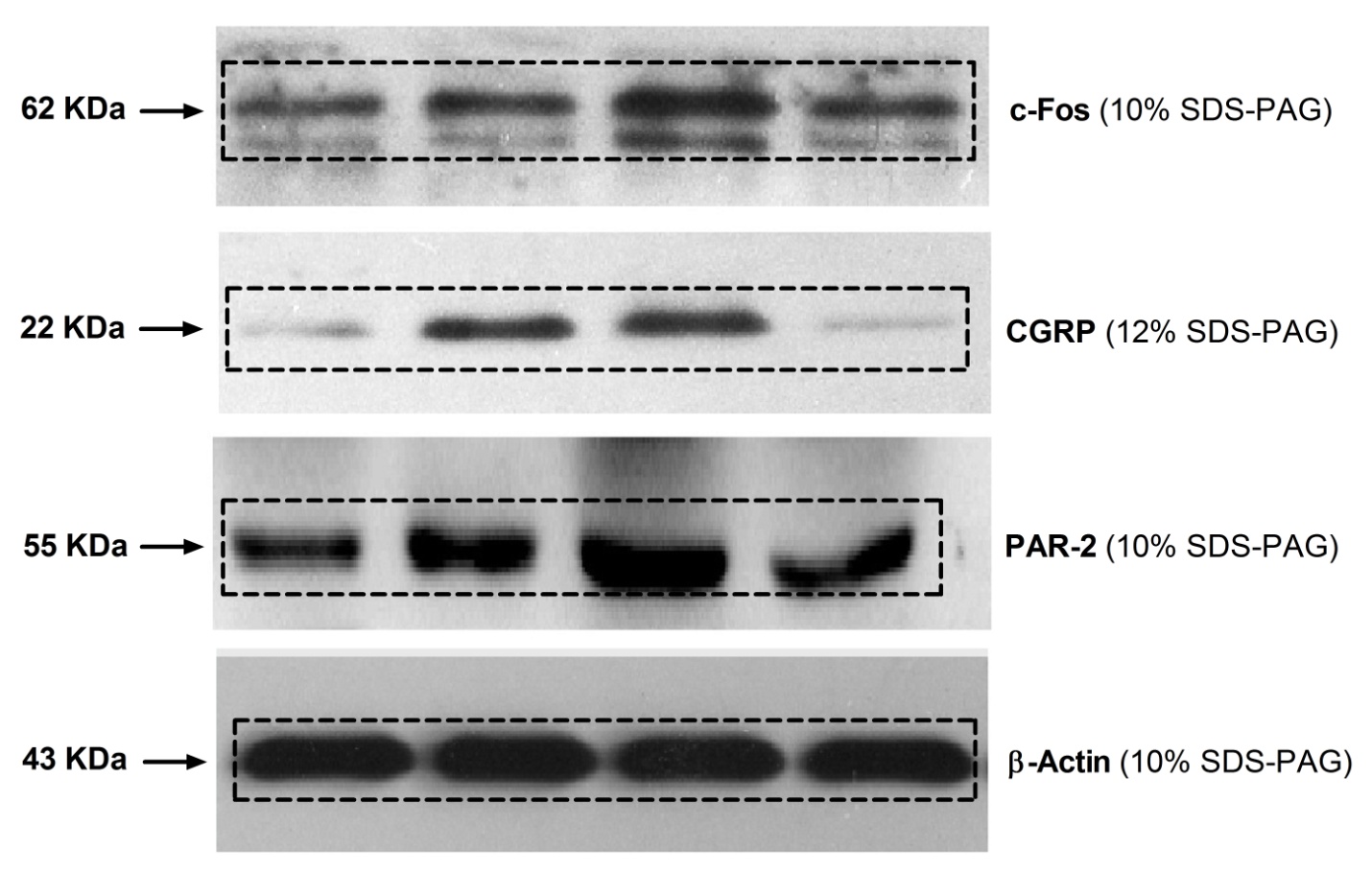


Supplementary Fig. S1. Full-length western blots for Figure 5a.
